# Prevalence of Child Maltreatment in Adolescents Attended At a Mental Health Day Hospital in Chile

**DOI:** 10.1192/j.eurpsy.2026.11158

**Published:** 2026-07-31

**Authors:** T. Ferrer, L. Blasco, I. Campusano-Castañeda

**Affiliations:** 1 Parc Sanitari Sant Joan de Déu, Sant Boi de Llobregat, Spain; 2 Salud Mental y Psiquiatría, Hospital Dr. Exequiel González Cortés, Santiago de Chile, Chile

## Abstract

**Introduction:**

Child maltreatment is highly prevalent worldwide and represents a major public health problem with long-lasting consequences. In Chile, 31.9% of adolescents reported lifetime sexual victimization, with higher rates among females (39.7%) compared to males (23.9%). Scientific literature on maltreatment in child and adolescent attended at mental health services in Chile is scarce, although international evidence suggests that these populations are more frequently exposed due to higher adversity and psychosocial risk conditions.

**Objectives:**

The aim of this study is to estimate the prevalence of child maltreatment (including violence and sexual abuse) among adolescents admitted to the Dr. Exequiel González Cortés Mental Health Day Hospital during a one-year period, from July 2024 to June 2025.

**Methods:**

This is a descriptive, retrospective study. Data were obtained from the hospital database, followed by statistical prevalence analysis using Excel platform.

**Results:**

Of the total sample (n=49), 61.22% of the adolescents attended were victims of violence, of whom 16.67% were male and 83.33% female. None of the patients acted as perpetrators. A total of 20.41% were victims of sexual abuse, with 10% being male and 90% female. Among these cases, 20 court reports were issued during treatment at the Day Hospital.

**Conclusions:**

The results show a high prevalence of child maltreatment, particularly among female adolescents treated at our Mental Health Day Hospital. These findings highlight the need for systematic trauma assessment in child and adolescent mental health services, as well as the development of specific intervention strategies.

**Disclosure of Interest:**

None Declared

